# The Human Disharmony Loop: The Anatomic Source Behind Subacromial Impingement and Pain

**DOI:** 10.3390/jcm14165650

**Published:** 2025-08-09

**Authors:** Ketan Sharma, Jaicharan Iyengar, James Friedman

**Affiliations:** 1 St Luke’s Plastic and Reconstructive Surgery, Boise, ID 83642, USA; 2 Alpine Orthopaedic Medical Group, Stockton, CA 95204, USA; jaicharan.iyengar@commonspirit.org (J.I.); james.friedman46@gmail.com (J.F.)

**Keywords:** subacromial impingement, shoulder pain, scapula, dyskinesia, pectoralis minor, weakness

## Abstract

**Background:** Subacromial impingement or pain syndrome (SAPS) is the most common diagnosis for chronic shoulder pain. Current surgeries do not reduce long-term pain, suggesting they miss the root etiology. Previously, we described the Human Disharmony Loop (HDL), where the unique lower trunk innervation to the pectoralis minor (PM) causes scapular dyskinesis and deforms its connections, including tugging the acromion down and impinging the subacromial structures. We hypothesize that SAPS patients who meet HDL criteria would benefit significantly from PM tenotomy with infraclavicular brachial plexus neurolysis (PM + ICN) alone. **Methods:** SAPS patients who met HDL diagnostic criteria were treated with PM + ICN, with secondary distal neurolysis if needed. Outcomes included pain and shoulder abduction ROM. Six-month follow-up minimum was required. **Results**: *N* = 140 patients were included. Median age was 49. Prior surgeries included 27% subacromial decompression/acromioplasty, 21% rotator cuff repair, 16% biceps tenodesis, 4% SLAP repair, 2% labral repair, 7% distal clavicle resection, 10% reverse total shoulder arthroplasty (rTSA), 1% rib resection with scalenectomy, 16% cervical spine fusion, 28% distal neurolysis. Median pain decreased from 8 to 2 and median shoulder ROM increased from 90 to 180 degrees. Positive impingement signs on exam decreased from 100% to 11%. (*p* < 0.01) **Conclusions**: In a large series of SAPS patients, evaluation and treatment for the HDL significantly reduced pain and restored motion. These findings suggest that in many patients SAPS may be a subset of the HDL: the ventral PM disturbing the scapula constitutes the anatomic basis and optimal surgical target behind SAPS.

## 1. Introduction

The most common reason patients present to a shoulder surgeon is chronic pain [1,2]. Worldwide, large proportions of the population suffer from shoulder pain on a daily to yearly basis [3]. One-third of the population endorses shoulder pain at any given point in time [4]. Shoulder pain is the third most common cause of disability [5,6]. Elite athletes are no exception [7].

The most common diagnosis given for shoulder pain is subacromial pain syndrome (SAPS) [8,9], previously known as impingement [10], constituting approximately 65% of visits to shoulder specialists [8]. Patients with SAPS present with anterior shoulder pain, radiating pain to the mid-arm, and pain/weakness with overhead reach chiefly during the 60–120 degree mid-arc of abduction [11]. Patients exhibit subacromial bursitis, tendinopathy or tears of the rotator cuff, and tendonitis of the bicipital tendon—the three structures within the subacromial space bounded by the overlying lateral acromion and underlying humeral head [1]. SAPS presents as a spectrum from bursitis alone to degenerative tendinopathy to frank cuff tears [12]. Surgery is generally advised after failure to prove despite 3–6 months of maximum conservative management [4]. SAPS is known to present commonly with other chronic pain syndromes centered at the shoulder, including scapular dyskinesis and pectoralis minor syndrome (PMS) [13]. Pectoralis minor syndrome (PMS) is described as infraclavicular compression neuropathy of the brachial plexus contributing to distal hand numbness/tingling and weakness [14]. Scapular dyskinesis refers to abnormal glide of the scapula along the thorax during shoulder motion, but whether it is a cause or effect of concomitant shoulder pathology remains uncertain [15]. 

The most common surgical treatments for SAPS include subacromial decompression or acromioplasty, bursectomy, CA ligament release, coplaning, and rotator cuff repair [4]. Acromioplasty in particular has increased in popularity recently [16]. However, high quality evidence repeatedly demonstrates these procedures do not reduce the chief complaint of shoulder pain or improve function [11,17,18,19,20,21]. Subacromial decompression provides no benefit over sham arthroscopy [22] or conservative management alone [9,18]. Acromioplasty adds no benefit to rotator cuff repair [23,24]. Even for symptomatic rotator cuff tears, whether surgical repair offers any long-term clinical benefit is uncertain [25]. Hence, some have contended SAPS is a “medical myth” and that “surgical treatment should have no role in the treatment of these patients” [11], while others propose to discard the term “impingement” altogether [21,26,27].

Thus, the contemporary state of the field can be summarized as follows: the most common surgical treatments performed by shoulder surgeons, for the most common diagnosis of SAPS, for the most common presenting complaint of shoulder pain, simply do not demonstrate efficacy.

Nonetheless, these patients are clearly suffering with a specific and reproducible set of shoulder symptoms [11], and demonstrate a predictable proximal etiology of inflammation and degeneration of anatomic structures in the subacromial space [9]. The ultimate etiology, however, remains unknown. Contentious debate continues between two predominant schools of thought [12]. The extrinsic hypothesis argues that the overlying anterior acromion, coracoacromial (CA) ligament, and acromioclavicular (AC) joint impinge and degrade the subacromial bursa, rotator cuff, and bicipital tendon [28]. The intrinsic hypothesis counters that inherent degradation of these structures due to diminished vascular supply, tensile forces, and/or aging first generates the inflammation [2]. Given the inefficacy for interventions that target the extrinsic sources, recent tides have turned in favor of the intrinsic proponents, but neither provides satisfactory causative explanations [2]. Yet many still favor the extrinsic hypothesis as “the surgical findings observed in the subacromial space during shoulder arthroscopy are hard to be explained if not for a contact between the rotator cuff and the acromion.” [9]. We conclude the true anatomic causes of SAPS, whether one or multiple, have yet to be described.

**Figure 1:**
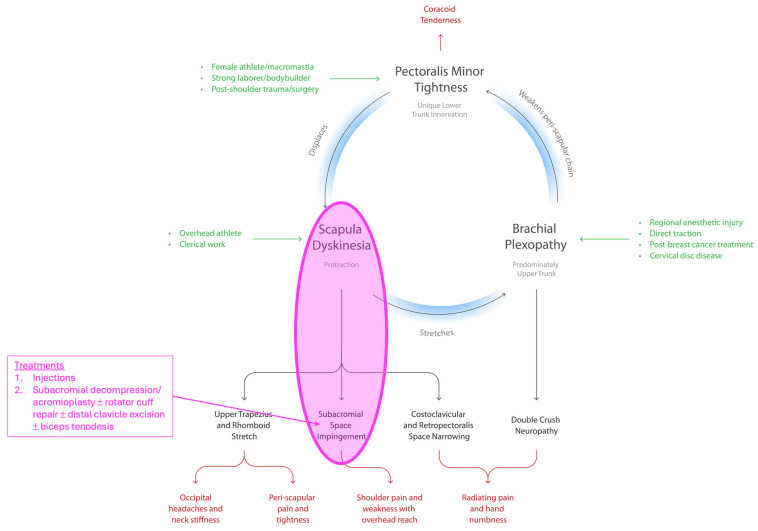
Subacromial Pain Syndrome (SAPS) as a Subset of the Human Disharmony Loop (HDL). The HDL is a clinical model of human upper limb pain centered on an unstable equilibrium around the scapula due to the unique lower trunk innervation to the pectoralis minor. The central loop has three elements, each causing anatomic sequelae. Diverse groups of patients can enter via each element, seen in green. The anatomic sequelae then produce coracoid tenderness and four distinct groups of clinical symptoms (bottom row). SAPS is the subset of the HDL where the pull of the PM pulls the acromion down, thereby impinging the subacromial structures, producing cuff tendinopathy, subacromial bursitis, bicipital tendonitis, and causing shoulder pain and weakness with overhead reach. Current treatments target a pathological effect and miss the true anatomic cause.

**Figure 2:**
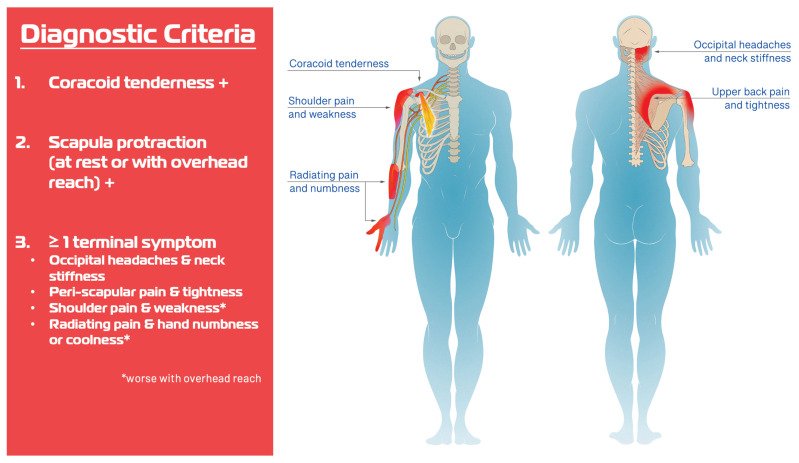
HDL Diagnosis. Diagnosis of HDL is based on two anatomic and one symptomatic criterion and is derived purely from history and physical exam.

Previously, we described the Human Disharmony Loop (HDL), a clinical model of upper limb pain centered at the shoulder [29]. (Figure 1) The scapula, which coordinates all function between the body (thorax) and arm (humerus), is controlled by the ventral pectoralis minor (PM) and dorsal peri-scapular muscles. The PM uniquely carries lower trunk innervation. This asymmetry generates an unstable equilibrium that predisposes the PM to overpower the dorsal peri-scapular muscles leading to scapular dyskinesia (protraction) [29]. One pathoanatomic sequelae includes lowering the acromion, narrowing the subacromial space, and impinging the bursa, cuff, and bicipital tendon. Three terminal symptoms include anterior shoulder pain, weakness with overhead reach, and radiating pain [29]. The HDL is diagnosed by strict anatomic and symptomatic criteria derived from history and physical exam [29] (Figure 2). Treatment for the HDL consists of ‘breaking the loop’ via tenotomy of the PM insertion off the coracoid followed by neurolysis of the infraclavicular brachial plexus (PM + ICN). In our first report, we observed that several intractable SAPS patients who had failed prior optimal surgical repair nonetheless improved drastically with near-complete elimination of pain and restoration of shoulder motion following HDL treatment. Hence, we suspect that the PM pulling down on the coracoid and disturbing scapula mechanics may constitute a hidden anatomic cause producing some forms of SAPS. Importantly, this would explain both the repeated intra-operative findings and the inefficacy of current surgeries. If true, a PM + ICN procedure would be expected to dramatically improve symptoms in patients suffering from SAPS. In this paper, we test this hypothesis and report the outcomes of PM + ICN alone to treat pain and weakness in SAPS patients who meet HDL criteria.

## 2. Materials and Methods

This is a prospective case series of patients treated at two sites by a uniform treatment protocol. All were evaluated by a fellowship-trained board-certified hand, sports, or shoulder surgeon. Inclusion criteria included: age > 18 years, a diagnosis of SAPS, and meeting HDL diagnostic criteria. (Figure 2) Exclusion criteria included: follow-up < 6 months. Patients were diagnosed with SAPS based on the presence of painful arc test and at least one positive Neer’s or Hawkin’s test. All patients trialed at least 3 months of therapy before being offered surgery. Patients were evaluated pre-operatively and at 2, 6, 12, and 24 weeks post-operatively. At each visit, patients completed a self-reported pain questionnaire. Shoulder abduction range of motion (ROM) values were measured independently by a trained and certified physical therapist at a separate evaluation without the surgeon present. Scapula dyskinesia was classified into four stages based on physical exam. A classification of none meant no protraction of the scapula at rest with the shoulder adducted or with overhead reach, dynamic meant no protraction at rest compared to the contralateral side but notable protraction when the patient attempted overhead reach. Static meant protraction at rest with the shoulder adducted and was subdivided into two categories: reversible and irreversible. With static reversible, the protracted scapula at rest could be manually reduced to normal position symmetric to the contralateral side by the examiner’s hand, whereas with static irreversible, the protracted scapula at rest could not be manually reduced. Each patient underwent open PM tenotomy with brachial plexus neurolysis using a previously published technique [29]. Specifically, a ~3 cm incision is made over the deltopectoral interval, the cephalic vein is protected, crossing vascular branches are ligated, the coracoid is palpated, and the conjoined tendon and PM insertion are identified. Carefully, the conjoined tendon is protected while the PM insertion is transected off the coracoid. Following this, the brachial plexus is identified deep to the fat pad and externally neurolysed from the clavicle down to the conjoined tendon. This was followed by a specific PT protocol. From weeks 1–4, shoulder motion was initiated but weight-bearing was limited to 3 lbs. or less to protect the incision. At week 4, nerve glides were started of the brachial plexus, axillary, radial, and median nerve. At week 6, strengthening was started of the upper trapezius and rhomboids along with scapular retraction postural training and taping if needed. The emphasis is to restrengthen scapula retraction once the deforming protraction force of the PM is eliminated. Patients were surveilled for residual neuropathy and offered secondary neurolysis at 3 months for neuropathic lesions causing lingering pain and/or weakness. Outcomes included pain and shoulder ROM in the abduction plane. Institutional Review Board (IRB) approval was obtained, and need for consent was waived as the data was anonymous and posed minimal risk to patients. Statistical analysis was performed using Student’s *t*-test and chi-squared analysis to compare continuous and categorical variables of interest, respectively, using STATA v19.0.

## 3. Results

*N* = 140 patients were included. Median age was 49. Sex was 41% male and 59% female. Prior surgical treatments included 27% subacromial decompression or acromioplasty, 21% rotator cuff repair, 16% biceps tenodesis, 4% SLAP repair, 2% labral repair, 7% distal clavicle resection, 10% reverse total shoulder arthroplasty (rTSA), 1% rib resection with scalenectomy, 16% cervical spine fusion, 28% distal neurolysis. Of the 52 patients with pre-operative MRIs, findings included 80% supraspinatus pathology, 4% subscapularis pathology, 17% bicipital tendonitis, 15% SLAP tear, 12% labral tear. Additionally, 88% of patients endorsed symptom relief with a medial retrocoracoid injection (Table 1).

Median preoperative pain was 8/10. Median shoulder abduction ROM was 90 degrees. Baseline scapular dyskinesia was 3% dynamic, 49% static reversible, 49% static irreversible. Six months postoperatively, median pain decreased to 2/10. Median shoulder ROM increased to 180 degrees, and presence of positive impingement signs on exam went from 100% to 11%. Post-operative scapular dyskinesia redistributed to 94% none, 6% dynamic, 0% static reversible, 0% static irreversible. All differences were statistically significant (*p* < 0.01). Additionally, 19% of patients required secondary neurolysis for the axillary (6%), radial (9%), ulnar (10%), and median (6%) nerves (Table 2).

## 4. Discussion

In this study, PM + ICN alone dramatically reduced pain and restored shoulder abduction in SAPS patients who met HDL criteria. Our findings support our hypothesis that some forms of SAPS may result from the pull of the PM on the coracoid from below, which tilts the acromion inferior and medial, narrows the subacromial space, and secondarily impinges the subacromial bursa, rotator cuff, and bicipital tendon. SAPS may itself be a subset of the Human Disharmony Loop (Figure 1).

**Figure 3:**
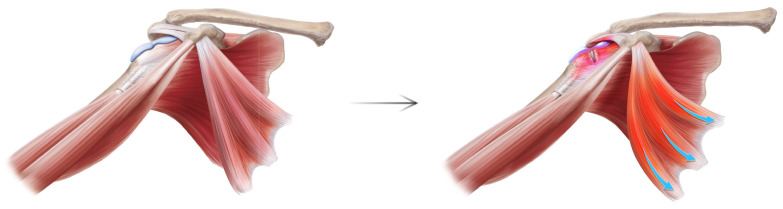
Subacromial Pain Syndrome Pathoanatomy: Frontal. On frontal view, the root pathological source is the deforming force from below of the PM tugging down onto the coracoid (blue arrow, right), which lowers the acromion and its associated structures, thereby impinging the subacromial space and producing subacromial bursitis, cuff tendinopathy to eventual tears, and bicipital tendonitis.

**Figure 4:**
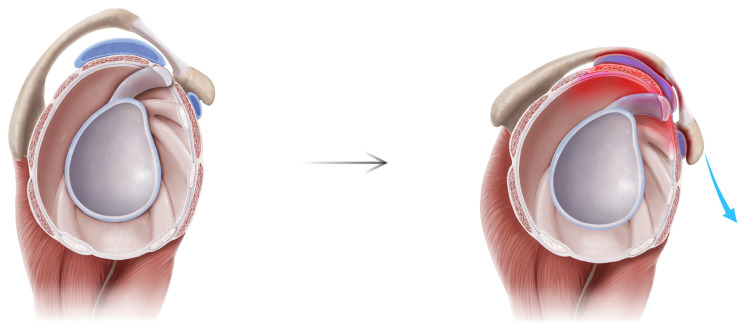
Subacromial Pain Syndrome Pathoanatomy: Sagittal. On sagittal view, the root pathological source is the deforming force from ventral of the PM tugging down onto the coracoid (blue arrow, right), which lowers the acromion and its associated structures, thereby impinging the subacromial space and producing subacromial bursitis, cuff tendinopathy to eventual tears, and bicipital tendonitis.

The HDL is a unifying chronic pain syndrome of the human upper limb. Due to the asymmetric C8-T1 lower trunk innervation to the PM, the human scapula is prone to dyskinesis. In almost all forms of dyskinesis, the scapula protracts [15]. Only the PM pulls the scapula in the direction of protraction, which is the combination of lateral translation, internal rotation, and anterior tilt. The protraction deforms the numerous connections of the scapula and pathologizes the full kinetic chain of the upper limb girdle. Previous explanations of SAPS have focused on rotator cuff dysfunction, which exerts a direct effect on the proximal humerus, and therefore can only displace the scapula indirectly through glenohumeral articular linkage. In contrast, the HDL provides a linear anatomic pathway: the PM pulls down on the coracoid which lowers the acromion, impinges the subacromial structures, inflames and degrades the bursa, cuff, and bicipital tendon, and produces the chief complaints of pain and overhead reach weakness (Figure 3 and Figure 4).

Anatomically, this explains the intra-operative observations seen repeatedly: subacromial bursitis, rotator cuff tendinopathy to eventual tears, bicipital tendonitis, and CA ligament deep surface fraying. But it also answers why current surgical interventions that target these supposed offending structures via subacromial decompression/acromioplasty, CA ligament debridement, and distal clavicle excision do not ameliorate pain or improve function—because they are also victims and not culprits. The root source lies below the subacromial space itself. (Figure 3 and Figure 4) Contemporary debate has raged between the many alleged extrinsic (acromion morphology, glenohumeral instability, AC joint degeneration, CA ligament degeneration, coracoid impingement) versus intrinsic (muscle weakness, shoulder overuse, degenerative tendinopathy) causes of SAPS. However, both suffer from incomplete mechanistic explanations [2] and invoke a myriad of proximate causes which are themselves left unexplained. MRIs show the pathological sequelae within the subacromial space which then become surgical targets, but not the pathological cause.

Previously, the causative relationships between the entities of PM tightness, scapular dyskinesis, and shoulder impingement has remained clouded in uncertainty and baffled surgeons [13]. PM tightness is traditionally thought to occur with impingement and cuff pathology [30] but develop after abnormal scapulothoracic motion [31,32]. Thus, while PM release has certainly been shown to improve scapular mechanics, widen the subacromial space, and decrease pain [33,34,35], “the cause of PM tightness is not fully understood” [34]. In contrast, the HDL uniquely identifies a single anatomic source as the underlying cause of scapular dyskinesis [29] and then clarifies a clear causation pathway between PM tightness, scapular dyskinesis, and subacromial impingement (Table 3). Hence, the HDL accounts for all chronic pain symptoms associated with SAPS including headaches, impingement, and neuropathy, and better identifies a broader set of patients who will benefit from PM + ICN.

**Figure 5:**
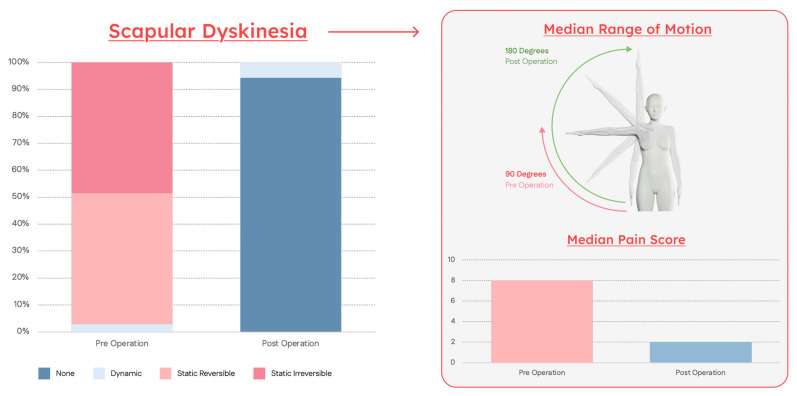
Normalization of Scapular Dyskinesia Produces Improvements in Range of Motion and Pain. Normalization of scapular dyskinesia following PM tenotomy produced full restoration of ROM and significant reduction in pain, demonstrating that the root cause underlying subacromial impingement and pain is neither extrinsic nor intrinsic but scapular.

The HDL potentially demystifies the century-long uncertainty: true cause of subacromial impingement may be neither extrinsic nor intrinsic but scapular. The results of our study—significant reductions in pain with near-full restoration of shoulder abduction—followed normalization of scapular kinesis, supporting this conclusion. (Figure 5) The prior surgical history and MRI findings of patients in the study further challenge existing models of SAPS. Many patients had prior subacromial decompressions, cuff repair, biceps tenodesis, and distal clavicle excision, but with enduring pain and weakness. Furthermore, those with preoperative MRIs displayed anatomic findings predicted by the HDL pathway: supraspinatus tendinopathy or tears, bicipital tendonitis, and SLAP tears. (Figure 3 and Figure 4).

Other mysteries relating to SAPS may be answered by the HDL. Radiating neuropathy is another HDL symptom, and is frequently seen in SAPS [29]. Scapula protraction is observed in SAPS patients [11,36]. Patients who demonstrate impingement on exam also exhibit both a protracted scapula and dorsal peri-scapular muscle weakness [37,38], two key HDL elements. Exercises that strength the dorsal peri-scapular stabilizers and reverse protraction of the scapula also reduce the need for surgery [39]. Overhead sports athletes with shoulder pain demonstrate a shorter and tighter PM on exam [40,41,42]. SAPS patients exhibit a significantly more active PM than asymptomatic patients when performing overhead shoulder elevation, while the other peri-scapular muscles remain similar [43]. Stabilization of the scapula reduces pain and disability in SAPS patients [44]. Scapular dyskinesis produces shoulder pain in athletes through secondary pathologic effects on the biceps-labrum complex [45]. Prior investigations have missed the underlying etiology, including: acromion morphology [46] does not correlate with chronic impingement [47], subacromial steroid injection is equally effective as acupuncture in pain reduction [48], neither presence of a rotator cuff tear nor acromiohumeral distance correlates with pain [11,49], physical examination tests for impingement suffer from poor diagnostic accuracy [50]. Each of these previous findings can be explained in light of the HDL model.

This study suffers from several limitations. As a non-randomized case series limited to two sites, our results must be replicated before establishing generalizability and causality. SAPS was diagnosed based on a combination of two clinical criteria, although no gold standard exists [9]. Our results only apply to SAPS patients who meet HDL criteria, and future studies need to investigate the relationship between the HDL and internal or subcoracoid impingement. Importantly, the need for secondary neurolysis in 19% of patients suggests that SAPS occurs concomitantly with distal neuropathy consistent with the well-known “double crush” phenomenon. Notably, 11% of patients exhibited continued impingement after treatment, showing that isolated impingement can persist, albeit uncommonly. We advocate ‘breaking the loop’ first, surveying for residual neuropathy or impingement, and addressing lingering pain generators later, only if they remain bothersome to the patient. Our principal outcome of self-reported pain is subjective, although no truly objective measurement exists, but nonetheless significant reductions in self-reported pain in a historically unsolvable chronic pain population can be meaningful. Our 6-month outcomes do not prove durability of results, and we do intend to report longer follow-up in subsequent studies. Future studies should utilize a consensus diagnostic standard and randomize PM tenotomy versus standard of care in large multi-institutional trials to demonstrate causality and generalizability of our study findings.

Nonetheless, in a large series of SAPS patients, PM + ICN (and secondary distal neurolysis if necessary) predictably and dramatically reduced pain and restored motion. We propose that some forms of SAPS may be a subset of the HDL: the ventral PM disturbing the scapula constitutes the anatomic basis and optimal surgical target in this historically challenging chronic pain patient population.

## Figures and Tables

**Table 1:** Patient Characteristics.

Variable	*N* = 140
Age	49 [37–60]
Sex	Male 58 (41%)
	Female 82 (59%)
BMI	29 [25–33]
Workers Compensation	35 (25%)
Surgical History	
Subacromial decompression	38 (27%)
Rotator cuff repair	29 (21%)
Biceps tenodesis	23 (16%)
SLAP repair	5 (4%)
Labral repair	3 (2%)
Bankart repair	2 (2%)
Distal clavicle resection	10 (7%)
Clavicle ORIF	1 (1%)
Reverse total shoulder arthroplasty	14 (10%)
1st rib resection + scalenectomy	2 (1%)
Cervical spine fusion	22 (16%)
Distal neurolysis (carpal, cubital)	39 (28%)
MRI Findings	(*n* = 52)
Supraspinatus tendinopathy or tear	42 (81%)
Subscapularis tendinopathy or tear	2 (4%)
Bicipital tendonitis	9 (17%)
SLAP tear	8 (15%)
Labral tear	6 (12%)
Laterality	Right 80 (57%)
	Left 60 (43%)
Hand Dominance	Right 114 (81%)
	Left 26 (19%)
Medial Coracoid Injection	Provided Relief 99 (88%)

Median with [inter-quartile range].

**Table 2:** Clinical Outcomes.

Symptom	Preoperative	Postoperative ^1^	*p*-Value
Pain	8 [6–9]	2 [0–3]	<0.01
Scapular Dyskinesia Stage			
Stage I	0 (0%)	132 (94%)	
Stage II	4 (3%)	8 (6%)	<0.01
Stage III	68 (49%)	0 (0%)	
Stage IV	68 (49%)	0 (0%)	
Shoulder Abduction ROM	90 [90–100]	180 [180–180]	<0.01
Positive Impingement Signs	140 (100%)	15 (11%)	<0.01
Neuropathic Lesions ^2^			
Scalene muscles	86 (61%)	3 (2%)	
Suprascapular notch	88 (63%)	0 (0%)	
Quadrilateral space	127 (91%)	17 (12%)	<0.01
Radial tunnel	96 (69%)	28 (20%)	
Cubital tunnel	37 (26%)	31 (22%)	
Carpal tunnel	72 (51%)	33 (24%)	
Secondary Neurolysis ^3^		27 (19%)	
Suprascapular		0 (0%)	
Quadrilateral space	N/A	9 (6%)	N/A
Radial		13 (9%)	
Cubital		14 (10%)	
Carpal		9 (6%)	

^1^ Postoperative results are at the most recent 6-month time point.; ^2^ Neuropathic lesions were considered positive with a positive scratch-collapse test at each anatomic location; ^3^ Patients were surveilled for residual neuropathy and offered secondary neurolysis at 3 months for persistent symptoms.

**Table 3:** Key Differences between Pectoralis Minor Syndrome (PMS) versus Human Disharmony Loop (HDL).

	PMS	HDL
Etiology	Unknown	Unique asymmetric lower trunk innervation
Mechanism	Compressive neuropathy	Deformation of scapula
Symptoms	Distal neuropathy only	All: headaches, neck pain, shoulder impingement, myofascial trigger points, and distal and proximal neuropathy
Anatomic Relationships to Other Chronic Pain Entities	Completely unknown	Clearly specifies cause and effect
Prognostic Value	Minimal	Strong
Epidemiology	Very rare	Ubiquitous
Relative Importance in Upper Limb Chronic Pain	After-thought	Central

PMS and the HDL are extremely distinct clinical syndromes whose critical differences are highlighted here. In summary, unlike PMS, the HDL can (1) explain the etiology of PM tightness, (2) account for all symptoms seen and not just distal neuropathy, and (3) specify clear casual pathways with subacromial pain syndrome and scapular dyskinesis.

## Data Availability

The original contributions presented in this study are included in the article. Further inquiries can be directed to the corresponding author.

## Abbreviations

The following abbreviations are used in this manuscript:

PM	Pectoralis minor
SAPS	Subacromial pain syndrome
HDL	Human disharmony loop
MRI	Magnetic Resonance Imaging
ROM	Range of motion
CA	Coraco-acromial
AC	Acromio-clavicular
IRB	Institutional Review Board
TSA	Total shoulder arthroplasty
SLAP	Superior Labrum Anterior–Posterior
